# Regional Anesthesia to Save the Day for Kids: A Narrative Review of Literature About the Blocks to Know for Common Pediatric Surgeries

**DOI:** 10.3390/medicina62010162

**Published:** 2026-01-13

**Authors:** Hadi Ufuk Yörükoğlu, Can Aksu, Nur Nazire Yucal, Sevim Cesur, Alparslan Kuş

**Affiliations:** 1 Department of Anesthesiology and Reanimation, Faculty of Medicine, Kocaeli University, Kocaeli 41001, Türkiye; ufukyorukoglu@gmail.com (H.U.Y.); dr.aksu@gmail.com (C.A.); svmcsr@gmail.com (S.C.); alparslankus@gmail.com (A.K.); 2 Department of Anesthesiology and Reanimation, Başakşehir Çam & Sakura City Hospital, Istanbul 34480, Türkiye

**Keywords:** pediatric population, postoperative pain, regional anesthesia, pediatric pain management

## Abstract

Postoperative pain management in pediatric patients remains a significant challenge despite improvements in perioperative care. Regional anesthesia techniques applied as part of multimodal analgesia strategies offer the potential to reduce opioid use, accelerate recovery, and minimize side effects such as respiratory depression, nausea, and delayed mobilization. This review examines the clinical applications, advantages, and limitations of regional anesthesia blocks in the context of common pediatric surgical procedures—appendectomy, inguinal hernia repair, circumcision, cholecystectomy, and pyloromyotomy. We provide procedural comparisons in terms of analgesic efficacy, opioid-sparing effects and suitability for ambulatory surgery. In conclusion, regional anesthesia techniques have significant potential to improve postoperative outcomes in pediatric patients. However, block selection should be individualized, considering the type of surgical procedure, patient characteristics, and operator experience. Increasing applicability and routinely implementing ultrasound-guided procedures will encourage the safer and more effective use of these techniques in pediatric anesthesia.

## 1. Introduction

Significant advances have recently been achieved in pediatric surgery due to the spread of minimally invasive approaches and increased sensitivity to patient safety [1]. According to USA data, the number of same-day surgical procedures in the pediatric population rose by 50% between 1996 and 2006 [2]. The most common operations in the pediatric age group are post-fracture extremity surgeries, appendectomy, inguinal hernia repair, circumcision, pyloromyotomy, and cholecystectomy [3,4,5]. However, there is no standardization for postoperative analgesia management, which is still inadequate [6]. Inadequate pain control in same-day surgery may be explained by lack of trust in opioid use or insufficient analgesic use [7]. The use of regional techniques in pain management in the pediatric population can establish standardization in terms of analgesic use due to a short operative time and the prevention of complications associated with opioid use [2].

In addition to delaying physical healing, failure to establish postoperative pain control in children can also produce psychological effects in the long term, such as anxiety, sleep disorders, and behavioral changes [8]. In a multi-center study of 570 patients from 2017, in-hospital pain was rated as ‘severe’ across all age groups [9].

The pharmokinetic effects of systemic analgesic drugs vary with age, and the use of opioids, in particular, entails risks including respiratory depression, vomiting, and sedation. These represent some of the negative aspects of pharmacological control of postoperative pain in this patient population [10]. Regional anesthesia techniques provide effective pain control in both the intra- and postoperative periods, and at the same time constitute a safe and effective component of multimodal analgesia by reducing opioid requirements [11,12].

An up to date evaluation, supported by the current literature, of the efficacy, reliability, and ease of application of regional anesthesia techniques in children is of great important in terms of clinical decision-making processes. This narrative review is based on a targeted evaluation of the current literature, including key guidelines and peer-reviewed studies focusing on pediatric regional anesthesia. The literature search primarily included terms such as “pediatric regional anesthesia,” “postoperative pain,” “fascial plane blocks,” and “ultrasound-guided blocks,” with an emphasis on commonly performed pediatric surgical procedures. The purpose of this review is to facilitate improved perioperative pain management by summarizing the regional anesthesia techniques most commonly encountered in pediatric surgery in the light of the current literature, and thus to ‘save the day’ for children undergoing surgery. To ensure a comprehensive narrative review of regional anesthesia techniques used in common pediatric surgeries, a targeted literature search was conducted using PubMed, Embase, and Google Scholar. Medical Subject Headings (MeSH) and free-text terms related to the pediatric population, regional anesthesia, ultrasound-guided nerve blocks, and postoperative pain were used. The review focused on English-language studies addressing the efficacy, safety, and technical aspects of these techniques in pediatric patients.

## 2. Surgeries and Regional Anesthesia Techniques

### 2.1. Limb Fracture Surgeries

Childhood traumas are ubiquitous, and fractures represent 10–25% of such injuries [13]. An annual incidence of fractures of 20.2 per 1000 children has been reported [14]. Some of the most frequently encountered are fractures of the long bones of the upper (a higher incidence) and lower extremities [15]. According to Drendel et al., the most severe post-fracture pain in children is experienced in the first 48 h [16]. In addition, varying levels of pain have been reported in more than 75% of children with extremity fractures, and this pain has frequently been shown to be inadequately controlled [17]. Groenewald et al. reported that 50% of children undergoing invasive surgery (such as osteotomy, open reduction, amputation, and internal fixation) experience moderate-severe pain [18]. In general terms, pediatric orthopedic surgery frequently leads to severe postoperative pain, and this generally necessitates hospitalization and parenteral opioid use [19]. This pain developing following orthopedic interventions can significantly affect patients’ postoperative recovery. Merella and Mossetti reported an increased incidence of ultrasound-guided regional techniques for upper and lower extremity fractures in children, and suggested that these can be effective in preventing acute and chronic pain [20].

Surgery in cases of upper extremity fractures frequently involves the humerus, radius-ulna, and elbow. A study from 2019 reported that postoperative pain in children operated due to supracondylar humerus fracture peaked on the morning following surgery, and that pain only decreased to clinically insignificant levels on the third day [21]. Brachial plexus blocks are frequently employed as a component of postoperative multimodal analgesia in the upper extremity [22]. Depending on the location and type of surgery, brachial plexus block can be applied with different techniques, such as interscalene, supraclavicular, infraclavicular, and axillary blocks.

A case series by Amiri and Espandar reported that ultrasound-guided brachial plexus block provided long-term analgesia and represented a safe anesthesia alternative in children undergoing upper extremity surgery [23]. However, the importance of the experience of the practitioner was stressed due to the proximity of the supraclavicular region to the pleura and the presence of several important surrounding anatomical structures.

Infraclavicular block indications are similar to those for the supraclavicular block. A randomized study from 2021 compared pain scores between two groups following closed reduction with infraclavicular block and sedoanalgesia in patients admitted to the emergency department with forearm fractures. Pain scores were significantly lower in the group undergoing infraclavicular block, and parental satisfaction was also higher in that group [24]. The use of both blocks has been supported in clinical practice in the pediatric population. A randomized study involving 80 children revealed that both supraclavicular and infraclavicular blocks provided effective surgical analgesia [25].

Once infraclavicular block had assumed its place in pediatric anesthesia in clinical practice, various approaches were compared in terms of safe application and analgesic effect. Yayık et al. compared the lateral sagittal infraclavicular and costoclavicular approaches, reporting similar analgesic efficacy, but that the costoclavicular method exhibited a shorter block performance [26]. Güzel et al. compared supraclavicular and costoclavicular blocks. Those authors reported that although neither exhibited any superiority in terms of pain management, costoclavicular block was more practical, and that due to its shorter block performance it could be regarded as a good option in upper extremity surgery [27]. In the light of these findings, the costoclavicular block technique represents a more effective and safe alternative in pediatric patients when applied in the anatomical area where the brachial plexus can be seen in compact form at ultrasonography.

Regional anesthesia techniques also exhibit proven efficacy in the management of postoperative analgesia in lower extremity fractures (such as the femur, tibia, and ankle) [28]. Turner et al. compared durations of analgesia and analgesic drug requirements in patients with and without femoral nerve block due to femur fracture. The authors reported a longer duration of analgesia and lower analgesic drug requirements in the femoral nerve block group [29].

Advances in the use of ultrasound have facilitated clearer visualization of anatomical structures and observation of the spread of local anesthetic. This has expanded the range of regional anesthesia techniques and attracted particular attention to fascial plan blocks. One such, fascia iliaca compartment block has begun being increasingly frequently used for analgesia in femur fractures. Neubrand et al. compared pain scores and systemic analgesic use doses in patients who underwent fascia iliaca compartment block to control pain associated with femur fracture and in those who received systemic analgesics only. Both were significantly lower in the block group [30].

The fact that femoral block and fascia iliaca compartment block are safe and easily applied in the pediatric population is increasing their use in clinical practice. However, both blocks have some limitations for surgical procedures considering the post-application dermatome area. For example, it should be remembered that the block spread following infrainguinal fascia iliaca nerve block may be limited to the femoral nerve, and analgesia may be insufficient in the region innervated by the obturator nerve. For the same reason, femoral nerve block applied alone in large bone fractures may not be adequate in surgical procedures. The particular surgical procedure should be taken into account when planning for postoperative analgesia, considering block combinations or the use of blocks that also involve the obturator nerve, such as suprainguinal fascia iliaca compartment block and direct lumbar plexus block.

Villalobos et al. performed a retrospective study of children undergoing hip surgery in 2019 and compared lumbar plexus and caudal epidural blocks in terms of postoperative and intraoperative opioid consumption, and observed no significant difference between the two techniques [31]. Those authors emphasized that although the two blocks exhibit similar analgesic efficacy, lumbar plexus block is clinically superior due to its unilateral effect, longer duration of analgesia, and low urine retention risk. However, since this block is deeper and requires experience, its practitioner-dependent success rates restrict its widespread use [32].

Despite the effectiveness of regional techniques, a number of factors limit their use in pediatric fracture surgery. The limited effect duration of single-dose block procedures means that pain control is difficult to achieve, particularly in the later hours postoperatively. The establishment of protocols with continuous techniques with catheter installation may help optimize pain control under those conditions.

### 2.2. Cholecystectomy

The incidence of gallstone disease in childhood is increasing. Factors such as the increased prevalence of obesity, the growing use of total parental nutrition, and the early diagnosis of hemolytic diseases have all played a role in that increase [33]. Laparoscopic cholecystectomy is regarded as the gold standard surgical method in the treatment of gallstone disease in the pediatric age group [34]. Postoperative pain following laparoscopic cholecystectomy in children generally includes shoulder pain (referred pain) in addition to both visceral and somatic components. If not well treated, this postoperative pain can prolong hospital stays, delay early mobilization, and increase opioid requirements. Effective analgesic strategies that reduce opioid use to a minimum in the pediatric population are therefore of great importance. A retrospective quality improvement study evaluated 511 children who underwent laparoscopic cholecystectomy; intravenous ketorolac was most frequently employed in the group in which opioids were not prescribed, as a result of which the mean opioid dose decreased and the number of ‘telephone interviews’ performed for pain follow-up also declined [35]. A 27% decrease in analgesic consumption, lower prevalences of nausea-vomiting and pruritus, and a shortening of hospital stays were observed in the 32 month period following the application of the Enhanced Recovery After Surgery (ERAS) protocol in the quality improvement project conducted in 2015 [36]. These findings suggest that multimodal analgesia strategies that reduce opioid consumption in pediatric laparoscopic cholecystectomy contribute to both pain control and also to the more effective use of hospital resources. The use of regional anesthesia techniques in addition to systemic analgesics for postoperative pain control is becoming increasingly widespread. A study from 2015 compared paravertebral blockade with incisional local anesthetic administration and reported that despite similar results in the postoperative period, intraoperative opioid use was lower in the paravertebral blockade group [37].

A randomized controlled study investigating the efficacy of erector spinae plane (ESP) compared ESP block applied at the bilateral L1 level with a control group. Pain scores and additional analgesic requirements were lower in the ESP group [38]. ESP has been shown to provide adequate analgesia in abdominal surgery. It is easier to apply and relatively safer than the paravertebral block, and local anesthetic is known to spread to more than one paravertebral space [39]. Similarly in another retrospective study, local anesthetic was shown to spread to an area of at least five dermatomes following ESP application in 164 patients, and the block may be effective for surgical incisions from T1 to L4 [40]. Once the effectiveness of the ESP block had been proved, studies also investigated the most effective application level for the surgical field. In a case report from 2018, ESP block was performed from the T9 level in a child of 11 undergoing laparoscopic cholecystectomy surgery, and low pain scores were obtained [41]. In another case, ESP block was applied at the T9 level in a pediatric patient due to undergo laparoscopic cholecystectomy, and low pain scores were also observed [42]. However, Karaca et al. introduced a modification at the block application level, applying bilateral ESP block from the T7 level in a four-patient case series; they reported low postoperative pain scores and no additional analgesia requirement [43]. Examination of the literature in general shows that local anesthetic can affect various anatomical areas, depending on the vertebral level at which the ESP block is applied, and that a wide craniocaudal spread can affect more than one dermatome area. Due to the insufficient nature of the literature concerning the spread of the ESP block in children, the lack of information on the subject has led to a shift toward adult studies. No definitive results have therefore been obtained concerning the level at which the block should be applied in the pediatric patient group.

Mannava et al. compared the transversus abdominis plane block, another technique used for the purpose of analgesia during these operations, with local anesthetic infiltration into the wound site. No difference was observed between the two group in terms of opioid requirements or pain scores [44]. However, studies of the efficacy of the ultrasound-guided oblique subcostal transversus abdominis plane (OSTAP) block for laparoscopic cholecystectomy have revealed effectiveness against somatic pain [45].

In conclusion, due to the scarcity of studies regarding fascial plane blocks in the pediatric patient group and potential variability in local anesthetic spread, the selection of regional techniques should be based on the preference and clinical experience of the practitioner.

### 2.3. Pyloromyotomy

Pyloric stenosis is a congenital cause of gastrointestinal obstruction seen in the first weeks of life and generally characterized by progressive vomiting [46]. Ramstedt pyloromyotomy, the most frequently performed procedure in the treatment of this condition, is generally carried out laparoscopically. Despite being minimally invasive, skin incisions and stretching of the abdominal wall can still cause somatic pain. Visceral stimuli caused by pneumoperitoneum can also contribute to pain. Research shows that this postoperative pain is generally mild to moderate, but can also give rise to opioid requirements in some situations, particularly in the first hours. The establishment of appropriate pain control following surgery is highly important in terms of both patient comfort and recovery. A retrospective, multi-center cohort analysis examining 11,008 infants observed opioid requirements in 2842 (26%). The spread of opioid use, particularly to the day of surgery and after, resulted in an increase in hospital resource consumption and costs of up to 13% [47].

The importance of regional anesthesia techniques that reduce opioid requirements is enhanced by the possibility of opioid-use complications (such as respiratory depression, nausea-vomiting, and irritability), particularly in special patient populations such as newborns. Truncal blocks are some of the most preferred methods from that perspective.

The application of the OSTAP block in a newborn case report resulted in low postoperative Face, Legs, Activity, Cry, Consolability (FLACC) scores and a decreased opioid requirement [48]. This has been described as an alternative option in upper abdominal surgery due to its safety and ease of application, and, in contrast to transversus abdominis plane (TAP) blocks, its ability to provide visceral analgesia [49].

USG-guided rectus sheath blockade is another popular technique in this patient group in the literature. In their retrospective study, Kumar et al. compared local anesthetic infiltration and rectus sheath block in terms of postoperative analgesic efficacy and reported that the two exhibited similar analgesic effects [50]. However, one important advantage of rectus sheath blockade is that it provides effective analgesia using less local anesthetic compared to infiltration. The risk of local anesthetic systemic toxicity is higher in newborns in particular, for reasons such as immature hepatic metabolism, a lower protein binding capacity, and greater vascular absorption, and LA dose titration is therefore important [51].

Although the TAP and rectus sheath blocks provide effective analgesia for minimally invasive approaches, in the light of the visceral pain mechanism, they are known to be inadequate for major abdominal surgeries. Quadratus lumborum block (QLB), that is capable of affecting visceral pain, can therefore be applied as a good alternative. Despite the lack of powerful, randomized data specific to pyloromyotomy in the literature, it has been reported to provide effective analgesia in abdominal surgeries [52]. However, although it exhibits effective somatic and visceral analgesic efficacy, its use is restricted by its being technically more difficult and a deep block technique, and the fact that it requires practitioner experience.

Since pyloromyotomy is applied to the most vulnerable population of the pediatric age group, clinicians must choose between these regional techniques and plan effective multimodal analgesia management avoiding opioids, based on their knowledge and experience and the prevailing clinical conditions.

### 2.4. Appendectomy

Appendicitis is a common cause of acute abdominal pathology in childhood, and treatment frequently involves laparoscopic appendectomy. Both peritoneal inflammation and visceral and somatic pain are seen in children following this procedure. In their review study published in 2023, Alsharari et al. reported that postoperative pain was frequently observed in children following laparoscopic appendectomy. The average Visual Analog Scale (VAS) score in the first 24 h in that study was 4–5, and the pain decreased over time [53]. This analysis revealed that pain following laparoscopic surgery in child can reach moderate to severe levels, thus stressing the importance of multimodal analgesia strategies, reducing opioid consumption, and increasing patient comfort, particularly through the use of regional anesthesia techniques.

TAP block is one of the fascial plane blocks widely employed in children undergoing laparoscopic appendectomy. An approximately two-fold decrease in opioid consumption over 48 h (10.3 mg vs. 22.3 mg) was observed in children undergoing unilateral TAP block, and pain scores at rest and movement also decreased significantly [54].

Xiao et al.’s meta-analysis reported similar efficacy in TAP and caudal blocks, although additional analgesic requirements were lower in patients undergoing TAP block [55]. However, Sandeman et al. reported that bilateral TAP block application provided no significant advantage over a control group among patients who underwent port site local anesthetic infiltration. They also reported lower pain scores during ward follow-ups among patients who received the TAP block [56].

As we have already seen, since TAP block is ineffective in controlling visceral pain and may increase negative outcomes in pain management, particularly in laparoscopic procedures in which multiple trocars are placed on the midline, since it is unable to provide adequate analgesia. The use of regional techniques, such as erector spinae plane (ESP) block and quadratus lumborum block (QLB), with their broad field of effect among the fascial plane blocks, is therefore important. Although limited pediatric data suggest that the erector spinae plane (ESP) block provides both somatic and visceral analgesia, evidence in pediatric appendectomy remains limited. A study of 63 children undergoing lower abdominal surgery compared an ESP group and a caudal group. The ESP registered lower FLACC scores and longer-lasting analgesia [57].

QLB is anatomically deeper, thus providing more pronounced visceral analgesia due to the spread of local anesthetic to deeper areas via the psoas major and thoracolumbar fascia. It is regarded as superior, particularly in suppressing pain associated with peritoneal irritation, and is especially advantageous in surgery involving peritoneal manipulation, such as laparoscopic appendectomy. In the light of the areas of spread of QLB in cadaveric studies, it is thought to provide effective analgesia from T10 to L4 [58].

A study of 2033 pediatric patients, intended to evaluate the efficacy of the QLB in lower abdominal surgery compared to a control group, reported that this block can be safely used in children and entails low additional analgesic requirements [59]. In addition, a newly published meta-analysis determined greater postoperative analgesic efficacy and lower pain scores for QLB [60]. In a study involving a pediatric population undergoing laparoscopic appendectomy, Ellatif et al. compared the TAP block and QLB. Those authors revealed that QLB resulted in lower pain scores and lower opioid use. They also reported that the analgesic effect of QLB was longer than that of other regional techniques due to local anesthetic spread to the paravertebral area [61].

Different QLB types have been described, depending on the anatomical application site. These different forms of application are known to exhibit different clinical effects. For example, paravertebral spread has been shown to occur only with QLB. Cadaveric studies have also shown local anesthetic spread patterns and likely areas of effect in different QLB types [62].

The literature contains no specific recommendation regarding the selection of fascial plane blocks to be applied in appendectomy cases in the pediatric population. As with many other surgical procedures, regional technique selection should be based on the experience and clinical means of the surgeon.

### 2.5. Inguinal Hernia

Inguinal hernia is widely seen in the pediatric population, and 3% of all children are reported to require inguinal hernia repair [63]. Although the incidence is low, it is one of the most commonly encountered surgical pathologies in the pediatric population. Treatment generally consists of open or laparoscopic hernia repair within a same-day surgery program. Pain in both techniques is incisional and somatic, although it also contains visceral components. Effective analgesia in these cases is important in terms of early initiation of oral intake and patient satisfaction [64].

One of the most frequently applied techniques for optimal pain management following pediatric inguinal hernia repair is caudal epidural block. This is particularly widely employed in the repair of unilateral open hernias, and provides effective analgesia with high success rates. However, since this is a central technique, its potential disadvantages include a bilateral effect, the possibility of motor block, and the development of side-effects such as bladder dysfunction [65]. Due to these potential limitations attendant upon caudal block, alternative regional techniques have been investigated, with fascial plan blocks again coming to the fore.

Ilioinguinal/iliohypogastric nerve block provides effective intra- and postoperative analgesia by blocking the somatic nerves originating from the T12–L1 level in surgeries involving limited incisions in the inguinal region. It is thought to provide safe anesthesia in the critically ill population by using less local anesthetic under ultrasound guidance [66]. A retrospective study involving premature newborns reported that ilioinguinal/iliohypogastric nerve block combined with general anesthesia represents an alternative technique yielding effective analgesia in these high-risk patients [67]. Another study compared ilioinguinal/iliohypogastric nerve block and caudal block analgesia in unilateral inguinal hernia surgery and reported that the former involved lower pain scores and a similar duration of analgesia to causal block [68]. A lower motor block risk and earlier mobilization compared to caudal block increase the practicability of the ilioinguinal/iliohypogastric nerve block. However, due to anatomical variations and nerve structures running outside the plexus, this technique is also dependent on the experience of the practitioner.

TAP block, one of the oldest fascial plane blocks, is also widely employed for pain control in inguinal hernia surgery. This block targets the nerve branches between T7 and L1 in the abdominal anterior wall and is effective in the control of somatic pain. Since the II/IH nerves also course in this plane, TAP block applied with an appropriate technique can represent an alternative to the II/IH nerve block. Studies have compared the efficacy of the TAP block with control groups. With its lower pain scores and analgesia requirements, the TAP block has become a component of multimodal analgesia [69].

QLB is another block employed for this purpose. A randomized controlled study evaluating the postoperative efficacy of QLB following inguinal hernia repair reported lower analgesic requirements in the first 24 h and lower pain scores in the QLB group [70]. QLB targeting visceral and somatic analgesia may provide a long-term effect in postoperative pain.

In Guan et al.’s randomized, controlled study involving 120 pediatric patients undergoing inguinal surgery compared ESP and caudal blocks. The authors concluded that ESP block was superior in terms of postoperative analgesia and rescue analgesia [71]. ESP being a relatively peripheral technique compared to caudal block means that possible post-caudal block complications can be avoided. Although no complication rates were reported in that study, these differences described in the literature show that ESP block is a more reliable option. Increasing data in the literature suggest that combinations of these blocks or their use together with adjuvants can enhance their effectiveness [72].

Chronic pain is seen in approximately 10–14% of cases following inguinal hernia repair in adults [73]. However, there is a lack of data for the pediatric population. Nevertheless, due to the more limited use of opioid and regional techniques in pain management, the incidence of postoperative pain may be expected to be higher, and the development of chronic pain may therefore be more frequent. Multicenter studies are needed to establish the incidence of surgery-based chronic pain in this population.

### 2.6. Circumcision

Circumcision is a same-day surgical procedure performed for religious, cultural, or medical reasons [74]. Despite being brief in duration and minimally invasive, post-circumcision pain can result in severe restlessness, sleep disturbances, and delayed oral intake in the pediatric patient population. Controlling post-circumcision pain is highly important in terms of reducing psychological trauma, preventing complications, and increasing patient satisfaction. The literature shows that long-term behavioral changes and post-surgical anxiety may develop due to the severity of postoperative pain when effective analgesia is not established. In a prospective cohort study published in 2015, Zavras et al. showed that post-circumcision pain significantly exacerbated anxiety in children and was associated with behavioral changes based on data from 301 patients with a mean age of 7–8 years [75].

Dorsal penile nerve block (DPNB) and caudal block have been used in the control of post-circumcision pain for many years. These to regional techniques have frequently been compared in the literature and subjected to randomized, controlled studies and meta-analyses. Examination of these studies shows that both techniques provide effective analgesia. However, they differ significantly in terms of effect duration, application success, complications, and parental satisfaction. In a review study published in 2008, Cyna and Middleton observed no significant difference between caudal block and DPBN in terms of rescue analgesia requirements. However, side-effects such as motor function loss and leg weakness were more frequent in children subjected to caudal block [76]. More recent randomized studies have also yielded similar results. Another study reported a significant decrease in VAS pain scores in the first 1–3 h in a caudal block group, while that effect was more limited in the DPBN group. However, second day pain scores were similar between the two groups [77]. In parallel with this, a study from 2020 reported that DPNB might constitute a more appropriate alternative in circumcision surgery due to ease of application and low complication rates [78].

The literature suggests that DPNB is an alternative that provides safe and sufficient analgesia for circumcision procedures because the duration of action and analgesic effect are similar between it and caudal block, and that DPNB is easier to apply and safer in terms of its-side effect profile.

## 3. Conclusions

In conclusion, this review, a summary of the current literature, reveals the variety and efficacy of regional techniques in pediatric surgery, and their place in clinical practice. The blocks applied in the relevant surgeries and literature recommendations are summarized in Table 1. Postoperative pain management in pediatric surgery is of very great importance, not only in terms of the comfort of the pediatric population, but also of accelerating recovery, reducing complications, and preventing the development of chronic pain and psychological effects. Regional anesthesia techniques are currently becoming a standard component of safe and effective multimodal anesthesia strategies by reducing opioid requirements. Nevertheless, despite growing evidence supporting the efficacy of these techniques, their application in children requires particular attention to age-related anatomical and physiological differences. In pediatric regional anesthesia, careful selection of the local anesthetic type, concentration, and total dose is essential due to age-dependent pharmacokinetics and the risk of local anesthetic systemic toxicity (LAST), particularly in neonates and infants. Variations in pharmacokinetic parameters across pediatric age groups underscore the necessity of individualized block selection, careful dose calculation, and strict adherence to age-specific safety principles to minimize the risk of systemic toxicity, especially in neonates and infants. Children are more sensitive to local anesthetics due to reduced metabolic capacity and lower plasma protein levels, which result in decreased protein binding. Therefore, both the dose and concentration of local anesthetics should be reduced in pediatric patients [79].

**Figure 1:**
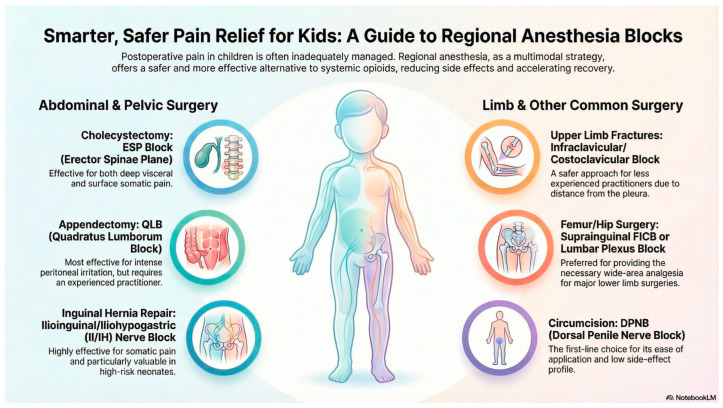
Smarter, safer pain relief for pediatric patients through regional anesthesia techniques. This infographic was generated using NotebookLM.

Previous studies frequently emphasize that block success largely depends on the experience of the practitioner, ultrasound use, and anatomical knowledge. Additionally, research into novel strategies such as block combination procedures or adjuvant drug use is growing. Future randomized, controlled studies and age-specific standard protocols will shed further light on the safety and efficacy of these techniques. This will thus help establish the most appropriate regional anesthesia method for each pediatric surgical procedure and improve the postoperative experience in pediatric patients (Figure 1).

### Key Messages

- Regional anesthesia provides effective, opioid-sparing postoperative analgesia in pediatric patients.
- Block choice should be individualized according to surgical procedure, anatomy, and clinician experience.
- Ultrasound-guided techniques increase block safety and accuracy.
- Fascial plane blocks (ESP, QLB, TAP, OSTAP) offer promising results for abdominal and pelvic pediatric surgeries.
- Incorporating regional anesthesia into multimodal analgesia protocols enhances comfort, reduces complications, and accelerates recovery in children.

## Figures and Tables

**Table 1:** Block types and doses according to surgery types.

Surgery Type	Block Types and Dose	Literature Recommendations
Appendectomy	ESP (0.3–0.5 mL/kg of 0.25% bupivacaine) QLB (0.2–0.4 mL/kg of 0.25% bupivacaine) TAP (0.3–0.5 mL/kg of 0.25% bupivacaine) Caudal Block (0.5 mL/kg of 0.25% bupivacaine)	– ESP or QL for both visceral and somatic involvement, TAP for somatic coverage, bilateral TAP may be considered in the presence of a midline incision.
Inguinal Hernia	Caudal Block (0.5 mL/kg of 0.25% bupivacaine) Ilioinguinal-Iliohypogastric (0.3 mL/kg of 0.5% bupivacaine) TAP (0.3–0.4 mL/kg of 0.25% bupivacaine) QLB (0.3–0.5 mL/kg of 0.25% bupivacaine) ESP (0.3–0.5 mL/kg of 0.25% bupivacaine)	– Caudal block covers a wider dermatomal area and entails a motor block risk; II/IH or TAP in situations in which caudal block is contraindicated, low-dose local anesthetic use and a low motor block risk.
Circumcision	Dorsal Penile Nerve Block (DPNB) (0.1 mL/kg of 0.25% bupivacaine) Caudal Block (0.5 mL/kg of 0.25% bupivacaine) Ring Block (Infiltration)	– DPNB or ring block can be used for analgesia for same-day surgery and ease of application.
Pyloromyotomy	Rectus Sheath Block (0.2 mL/kg of 0.25% bupivacaine) TAP (0.3–0.5 mL/kg of 0.25% bupivacaine)	– May be planned for preventing somatic pain and midline incisions.
Cholecystectomy	ESP Block (bilateral *) (0.3–0.5 mL/kg of 0.25% bupivacaine) TAP (0.2–0.3 mL/kg of 0.25% bupivacaine)	– ESP can be employed since it affects more than one dermatome area and spreads to the paravertebral area. – TAP can represent a component of multimodal analgesia due to somatic involvement and ease of application.
Upper Extremity Fracture Surgery	Brachial Plexus Block (Supraclavicular (0.15–0.2 mL/kg of 0.25–0.5% bupivacaine) Infraclavicular, Costoclavicular Block) (0.5 mL/kg of 0.25–0.5% bupivacaine)	– Infraclavicular block and supraclavicular block provide widespread and effective analgesia. – Costoclavicular block exhibits a shorter performance.
Lower Extremity Fracture Surgery	Femoral Nerve Block (0.2–0.5 mL/kg of 0.25% bupivacaine) Fascia Iliaca Block (0.5 mL/kg of 0.25% bupivacaine) Lumbar Plexus Block (0.2–0.5 mL/kg of 0.25% bupivacaine)	– Femoral block analgesia longer lasting. – It should be remembered that no obturator nerve involvement may occur with fascia iliaca block. – Lumbar plexus block has a unilateral effect, long-term analgesia.

The doses described apply to children older than 6 months. In infants younger than 6 months, a reduction in both the dose and concentration of local anesthetics is recommended. *: In bilateral block applications, reducing the concentration of the local anesthetic should be considered due to the potential risk of systemic toxicity.

## Data Availability

All data is available within the manuscript.
